# De-escalation of Sentinel Lymph Node Biopsy in Patients With Ductal Carcinoma In Situ

**DOI:** 10.7759/cureus.37383

**Published:** 2023-04-10

**Authors:** Hussain A Abdulla, Yasmeen Khalaf

**Affiliations:** 1 General Surgery, Salmaniya Medical Complex, Manama, BHR; 2 Surgery, Salmaniya Medical Complex, Manama, BHR

**Keywords:** breast cancer, omission of axillary surgery, management of axilla, ductal carcinoma in situ, sentinel lymph node biopsy

## Abstract

Introduction

Current guidelines recommend that sentinel lymph node biopsy (SLNB) be performed in patients with ductal carcinoma in situ (DCIS) undergoing mastectomy, in patients for whom the location of excision may compromise future SLNB, or if there is a high suspicion or risk of upstaging to invasive cancer on final pathology. Whether axillary surgery should be performed in patients with DCIS remains controversial. Our study aimed to examine the factors associated with the upgrade of DCIS to invasive cancer on final pathology and sentinel lymph node (SLN) metastases to evaluate whether axillary surgery may be safely omitted in DCIS.

Methods

Patients with a diagnosis of DCIS on core biopsy who underwent surgery with axillary staging between 2016 and 2022 were identified from our pathology database and retrospectively reviewed. Patients who underwent surgical management of DCIS without axillary staging and those treated for local recurrence were excluded.

Results

Out of 65 patients, 35.3% of patients were upstaged to the invasive disease on final pathology. 9.23% of cases had a positive SLNB. Predictive factors associated with upstaging to invasive cancer included palpable mass on clinical examination (P = 0.013), presence of a mass on preoperative imaging (P = 0.040), and estrogen receptor status (P = 0.036).

Conclusion

Our results support ongoing opportunities for the de-escalation of axillary surgery in patients with DCIS. In a subset of patients undergoing surgery for DCIS, SLNB may be omitted as the risk of upstaging to invasive cancer is low. Patients with a mass on clinical examination or imaging and negative estrogen receptor (ER) lesions have a higher risk of upstaging to invasive cancer, where a sentinel lymph node biopsy should be performed.

## Introduction

Ductal carcinoma in situ (DCIS) is a noninvasive type of breast cancer characterized by the abnormal proliferation of ductal epithelial cells confined by the basement membrane. In the setting of DCIS, there is no risk of potential spread to axillary lymph nodes or distant metastases. Most cases are detected as microcalcifications by screening mammography [1]. The vast majority of patients are diagnosed by core needle biopsy; however, there is a risk of underestimating the presence of invasive disease due to sampling error [2]. Approximately up to 40% of DCIS diagnosed by core needle biopsy are upstaged to invasive carcinoma on final surgical pathology, indicating the need for axillary staging through sentinel lymph node biopsy (SLNB) [3].

The National Comprehensive Cancer Network (NCCN) guidelines suggest that SLNB should be performed in patients with DCIS undergoing mastectomy or in those for whom the location of breast-conserving surgery (BCS) may compromise future SLNB [4]. While SLNB has relatively less morbidity compared to axillary dissection, it is still associated with complications such as lymphoedema, paraesthesias, and seroma [5]. There is a trend toward minimizing axillary surgery in clinically node-negative breast cancer patients, even in the presence of limited sentinel lymph node (SLN) metastases [6]. SLNB can also be safely avoided in elderly patients with early-stage, hormone-positive, and clinically node-negative diseases [7]. Several trials are investigating whether the omission of SLNB in clinically node-negative early breast cancer patients treated with BCS is oncologically safe [8]. If de-escalation of axillary surgery is considered safe in invasive breast cancer, this should be even true for cases of DCIS.

Identification of risk factors associated with upstaging invasive cancer and SLN metastases may allow us to evaluate whether SLNB is justified in patients with DCIS. This study aimed to assess predictors of upgrade to invasive disease and SLN positivity in patients undergoing surgery and SLNB for DCIS to determine whether it can be safely omitted.

## Materials and methods

Patients who had a preoperative diagnosis of DCIS on core biopsy and subsequently had axillary surgery performed between January 2016 and January 2023 were included in the study. Patients with ipsilateral breast tumor recurrence and those who underwent BCS without axillary intervention were excluded. All patients who underwent mastectomy had SLNB in the same operative setting. Some patients in whom the location of wide local excision may compromise future SLNB (for example, central, upper outer quadrant, or axillary tail lesions) underwent SLNB at the index operation. Otherwise, patients who underwent BCS and whose tumors were upstaged to the invasive disease on final pathology underwent delayed surgical axillary staging as a second procedure. Patients who met the inclusion criteria were identified from our pathology database (the pathology database of the Salmaniya Medical Complex, Manama, Bahrain). The data included age at diagnosis, tumor location, type of surgery, presence of an invasive component, nodal status, tumor grade, receptor status, multifocal or multicentric disease, and presence of a mass on clinical examination or preoperative imaging.

Our method of performing SLNB involves a dual technique, using blue dye and a radioactive tracer (Tc99). The intradermal injection of Tc99 was performed a few hours preoperatively on the day of surgery. After induction of general anesthesia, isosulfan blue dye was injected into the retroareolar region or intraparenchymal. Hand-held gamma probe guidance and visual inspection for blue dye were used to retrieve the sentinel nodes. We used an intraoperative frozen section, which was communicated to the operating surgeon by the pathologist within 45 minutes. Axillary dissection was performed only if macrometastasis was detected in more than two SLNs or with failure of SLNB.

Descriptive analyses were used to summarize the data and evaluate the clinicopathological variables between DCIS and upgraded invasive groups. To test for an association between the two groups, the Chi-square or Fisher’s exact test was used. Statistical analysis was performed using the IBM SPSS Statistics for Windows, Version 29.0 (Released 2022; IBM Corp., Armonk, New York, United States), and P values less than 0.05 were considered significant.

## Results

There were 65 eligible patients who had DCIS on a core biopsy and underwent surgery with SLNB (Table 1 and Table 2). All patients were female. The mean age was 52 years (range 25-93). 33 (50.7%) of cases were left-sided, with 39 (60%) of tumors localized to the upper outer quadrant. Eleven (16.9%) patients had multicentric diseases. BCS was performed in 23 (35.3%) patients, and 42 (64.6%) patients underwent a mastectomy. SLN identification was achieved in all but one patient who previously underwent BCS (98.4%). On final pathology, 23 (35.3%) patients had an upstaging of DCIS to invasive cancer, 16 to invasive ductal carcinoma, and seven to DCIS with microinvasion. Out of the patients that were upstaged, 14 had BCS, and nine patients underwent a mastectomy. SLNB was performed in the remaining nine patients even though there was no upstaging of DCIS to invasive cancer, as the location of the excision was thought to compromise future surgical axillary staging. Of the 42 patients with a final diagnosis of DCIS on surgical pathology, there were no cases of lymph node involvement. Six (9.23%) patients had a positive SLNB, one with micrometastasis and five with macrometastasis, at the time of mastectomy. Among patients with a final diagnosis of invasive cancer after BCS, there were no cases of SLN involvement. Axillary dissection was performed in six patients: five for macrometastasis and one for the failure of SLNB. Five of these patients had no further nodal metastases detected in the axillary specimen, indicating that the SLN was the only positive node. On final surgical pathology, patients who were upstaged to invasive cancer did not differ from those with DCIS with regard to age at diagnosis, tumor location, or extent of disease. Clinicopathological factors predictive of upstaging from DCIS to invasive carcinoma on final pathology included a palpable mass on clinical examination, the presence of a mass on preoperative imaging, and ER receptor status (Table 3).

**Table 1:** Patient clinical characteristics of the study population BCS: Breast-conserving surgery; LIQ: Lower inner quadrant;​​​​​​​ LOQ: Lower outer quadrant;​​​​​​​ UIQ: Upper inner quadrant; ​​​​​​​UOQ: Upper outer quadrant

Age	Number (Total = 65)
Mean	52
Median	50
Range	25-93
Tumor laterality	
Right breast	32
Left breast	33
Type of surgery	
BCS	23
Mastectomy	42
Tumor quadrant	
Central	13
LIQ	6
LOQ	6
UIQ	1
UOQ	39
Disease extent	
Unifocal	40
Multifocal	14
Multicentric	11
Palpable mass	
Present	38
Absent	27
Mass on preoperative imaging	
Present	40
Absent	25

**Table 2:** Tumor pathological parameters of the study population ER: Estrogen receptor; PR: Progesterone receptor; HER2: Human epidermal growth factor receptor 2;​​​​​​​ NA: Not applicable; ​​​​​​​DCIS: Ductal carcinoma in situ; ​​​​​​​IDC: Invasive ductal carcinoma

Tumor grade	Number (Total = 65)
Low	13
Intermediate	17
High	35
Comedo necrosis	
Present	31
Absent	27
ER status	
Positive	42
Negative	23
PR status	
Positive	38
Negative	27
HER2 status	
Positive	9
Negative	14
N/A	42
Final surgical pathology	
DCIS	42
DCIS with microinvasion	7
IDC	16
Nodal status	
N0	59
N1	5
N2	1

**Table 3:** Relationship between clinicopathological characteristics and risk of upstage to invasive disease ER: Estrogen receptor; PR: Progesterone receptor

Age	Upstage to invasive disease (in %)	P value
<50	12.3%	
≥50	23%	0.413
Tumor side		
Left	15.3%	
Right	20%	0.384
Palpable lesion		
Yes	30.7%	
No	9.23%	0.013
Mass on imaging		
Yes	27.6%	
No	7.69%	0.040
Multifocal disease		
Yes	10.7%	
No	24.6%	0.196
Multicentric disease		
Yes	9.23%	
No	26.1%	0.144
Comedo necrosis		
Yes	20%	
No	18.4%	0.987
Tumor grade		
Low	3.07%	
Intermediate	9.23%	
High	23%	0.209
ER status		
Positive	16.9%	
Negative	18.4%	0.036
PR status		
Positive	15.3%	
Negative	20%	0.092

## Discussion

In this study, we evaluated patients with DCIS diagnosed on core biopsy who subsequently underwent primary surgery and SLNB. We found the upstage rate of invasive cancer to be 35.3%, and this corresponds with the 8-48% range reported in the literature [9-13]. This variation can be due to the heterogeneity of DCIS and the interpretation of factors by the pathologist [10]. The rate of SLN positivity among these upstaged patients was 9.23%, and this is similar to what previous studies reported in the literature, ranging between 4-15% [3,9,13,14]. This low rate of SLN metastasis is clinically significant, suggesting the safety of delaying SLNB until a definitive diagnosis of invasive cancer is made [2].

There is no consensus in the literature regarding predicting factors for which DCIS tumors will be upstaged to invasive cancer, and preoperative identification of SLN metastasis is also difficult [15]. Previous studies have described several factors associated with the upstaging of DCIS to invasive disease, including young age, palpable mass on examination, mass on preoperative imaging, high-grade tumors, comedo necrosis, multicentric disease, and negative hormone receptor status [3,16,17]. In our cohort, upstaging was associated with a palpable mass, the presence of a mass on preoperative imaging, and negative estrogen receptor (ER) lesions. NCCN guidelines suggest that SLNB should not be performed during BCS but should be strongly considered if the patient with apparent pure DCIS is to be treated with mastectomy, in a patient for whom the location of BCS may compromise future SLNB (for example, upper outer quadrant or axillary tail lesions), or if there is a high suspicion or risk of the upgrade of the lesion on final pathology [4]. Criteria that have been associated with this higher risk of upstage include the presence of a mass on clinical examination or imaging, as in our study [18]. In our study, nine patients underwent surgical axillary staging for DCIS at the time of BCS because it was thought that the location of wide local excision may compromise future SLNB. These patients underwent unnecessary morbidity from axillary surgery, and therefore SLNB is discouraged during BCS. In the setting of BCS, second-stage SLNB is possible should the final surgical pathology reveal unexpected invasive cancer [3].

Hormone receptor-negative DCIS tumors have an increased risk of being upstaged to invasive carcinoma [17]. Although the prevalence of invasive cancer in our study was higher for hormone receptor-negative tumors than hormone receptor-positive tumors, the difference was only statistically significant for ER-negative tumors. Patients with hormone receptor-positive DCIS found to have the invasive disease can be treated with adjuvant hormonal therapy, as it confers a survival benefit, while adjuvant hormonal therapy is not indicated for hormone receptor-negative tumors [19]. Therefore, for ER-negative DCIS, routine SLNB during mastectomy is recommended, as SLN metastasis in these patients indicates the need for adjuvant chemotherapy [17].

In line with efforts towards de-escalation of axillary surgery in early breast cancer, ongoing trials are investigating whether SLNB contributes to staging or local control [8,18]. The SOUND trial is investigating whether physical examination and axillary ultrasound can diminish the need for SLNB in patients with BCS [20]. This has led to some authors advocating that routine SLNB in those undergoing mastectomy for DCIS is overtreatment and should only be reserved for patients with any uncertainty regarding the presence of invasive foci or a high risk of upstaging to invasive cancer, such as the presence of a mass on examination or imaging [13,14]. Although there have been reports of successful and technically feasible SLNB after mastectomy, index surgery is the best chance to assess axillary lymph nodes due to the disruption of lymphatic channels [18,21]. Recently, the SentiNot trial showed that marking the SLN with superparamagnetic iron oxide in patients with a preoperative diagnosis of DCIS resulted in avoiding upfront SLNB in 78.3% of patients, with delayed SLNB being feasible and yielding high detection rates once the invasive disease was found [22].

Our study has a few limitations. First, this is a retrospective study that was done at a single institution. However, the results of this study can be valuable to other centers that can build on these results and add to the literature. Another limitation is the relatively small sample size, although this is representative of the small number of SLNB being performed in patients with DCIS. Furthermore, we did not evaluate other predictive factors, such as the effect of the type of surgery, family history of breast cancer, and the presence of a breast cancer gene 1/breast cancer gene 2 (BRCA1/BRCA2) gene mutation. We could not analyze the effect of human epidermal growth factor receptor 2 (HER2) status on the risk of upstaging to invasive disease as it is not routine practice at our institution to test for HER2 receptor status in patients with DCIS.

## Conclusions

Our results support emerging evidence to de-escalate axillary surgery and minimize morbidity in DCIS. In the majority of patients undergoing mastectomy for DCIS, SLNB may be omitted as the risk of SLN positivity is low. Patients with the presence of a mass on clinical examination or preoperative imaging and ER-negative disease have a significant risk of upstaging to invasive carcinoma, and SLNB remains important to guide treatment decisions. For early breast cancer patients, it is likely that axillary ultrasound will replace the need for SLNB for axillary staging in the future. However, further larger-scale prospective studies are required to incorporate these findings in the axillary management of patients with DCIS. As per the long-established guideline recommendations, SLNB for DCIS is currently only warranted when a mastectomy is performed, although this may change in the future.
